# Optimising parameters for the differentiation of SH-SY5Y cells to study cell adhesion and cell migration

**DOI:** 10.1186/1756-0500-6-366

**Published:** 2013-09-11

**Authors:** Susan Dwane, Edel Durack, Patrick A Kiely

**Affiliations:** 1 Department of Life Sciences and Materials and Surface Science Institute, University of Limerick, Limerick, Ireland; 2 Stokes Institute, University of Limerick, Limerick, Ireland

**Keywords:** Cell migration, Cell adhesion, Neurite outgrowth, Focal adhesion kinase, SH-SY5Y cells

## Abstract

**Background:**

Cell migration is a fundamental biological process and has an important role in the developing brain by regulating a highly specific pattern of connections between nerve cells. Cell migration is required for axonal guidance and neurite outgrowth and involves a series of highly co-ordinated and overlapping signalling pathways. The non-receptor tyrosine kinase, Focal Adhesion Kinase (FAK) has an essential role in development and is the most highly expressed kinase in the developing CNS. FAK activity is essential for neuronal cell adhesion and migration.

**Results:**

The objective of this study was to optimise a protocol for the differentiation of the neuroblastoma cell line, SH-SY5Y. We determined the optimal extracellular matrix proteins and growth factor combinations required for the optimal differentiation of SH-SY5Y cells into neuronal-like cells and determined those conditions that induce the expression of FAK. It was confirmed that the cells were morphologically and biochemically differentiated when compared to undifferentiated cells. This is in direct contrast to commonly used differentiation methods that induce morphological differentiation but not biochemical differentiation.

**Conclusions:**

We conclude that we have optimised a protocol for the differentiation of SH-SY5Y cells that results in a cell population that is both morphologically and biochemically distinct from undifferentiated SH-SY5Y cells and has a distinct adhesion and spreading pattern and display extensive neurite outgrowth. This protocol will provide a neuronal model system for studying FAK activity during cell adhesion and migration events.

## Background

Brain development begins approximately three weeks after conception when the brain is composed of a single layer of flattened cells called the neural plate. A furrow forms from the front (rostral portion) to the back (caudal portion) of the neural plate to form the neural groove. This groove then begins to close to form the neural tube approximately 4 weeks after conception. The stem cells in the neural tube either undergo high levels of proliferation or migrate to their final location and differentiate to form the various nerve tissues. The nervous system uses neuronal migration to generate specific and intricate brain circuitries by positioning cell types from different origins in the same area. Neurons from the neural tube disperse throughout the central nervous system (CNS) by two main strategies; radial and tangential migration [1,2]. Radial migration involves migration of neurons in a perpendicular direction from the neural tube and these neurons often use radial glial fibres as a substrate to aid migration. In contrast, tangential migration involves migration of neurons parallel to the neural tube and these neurons do not require support to migrate (reviewed by Marin *et al.*[3]). Cell migration has an essential role in brain development as it not only establishes the formation of a highly specific pattern of connections between nerve cells but it is also required for axonal guidance and neurite outgrowth [4,5]. Neurite outgrowth during brain development is the mechanism by which neurons differentiate to grow neurites (developing axons and dendrites). The extension of an axon commonly involves travel across long distances and a process called pathfinding (a turning response to extracellular signals). Growth cones, the leading edge of neurites, are dynamic areas at the tip of an extending neurite. They are involved in the process of pathfinding during axonal extension and respond to attractive and repulsive cues. The non-receptor tyrosine kinase, FAK, is the most highly expressed kinase in the developing CNS and has an essential role in development. FAK has a role in neuronal migration and axonal guidance/neurite outgrowth [6,7]. FAK has been shown to be dispensable for tangential migration but required for radial migration as it regulates the assembly of Connexin-26 contact points between migrating cells and radial glial fibres [8]. Radial migration is the primary mechanism of neuronal migration for developing neurons which stresses the importance of FAK in the developing brain [9]. FAK has previously been reported to localise in the growth cone [10] and has been implicated in mediating the response to attractive and repulsive cues as growth cones migrate to their specific target (reviewed by Chacón and Fazzari [11]). FAK is important in axon outgrowth as it controls filopodia formation and actin nucleation through the phosphorylation of N-WASP [12]. High levels of FAK are expressed in the hippocampus and this protein has been shown to be important in long term memory storage [13-15]. FAK activity is essential for neuronal cell migration but the mechanisms regulating FAK in the developing nervous system are not well characterized and require further study.

An area of the brain that is commonly used for studying neurite outgrowth is the hippocampus. Unlike most other regions of the brain, a significant portion of hippocampal formation development occurs postnatally and is commonly used as a model in neurite outgrowth studies [16]. Hippocampal neurons are a good model system as they are continuously undergoing change due to their role in memory. However, it is difficult to manipulate these cells to characterise protein-protein interactions and commonly, neuronal-like cell lines are employed. SH-SY5Y cells are a human neuroblastoma cell line that are commonly used as a neuronal cell model in studies of Parkinson’s disease and Alzheimer’s disease as they can be differentiated to resemble dopaminergic neurons [17-20]. When cells are differentiated into neuronal-like cells, they stop proliferating, extend long neurites and express neuronal markers including GAP43 and β3-tubulin [21].

Ivankovic-Dikic *et al.*[22] reported that both adhesion and growth factor receptors should be stimulated simultaneously in order for neuronal cells to undergo optimal differentiation. FAK has been regarded as the convergence point of growth factor and integrin signalling for many years [23,24] and RACK1 has now been established as a scaffolding protein that mediates the converging of these signalling pathways [25,26]. The aim of our study was to further optimise a protocol for differentiating SH-SY5Y cells to monitor FAK activity during cell adhesion and migration events.

In this study, we show that SH-SY5Y cells differentiate well and express high levels of FAK when grown on laminin and in the presence of IGF-1. We confirm that this cell population are distinct from undifferentiated cells in both its adhesion and spreading profile and also confirm that when grown on laminin and in the presence of IGF-I that the cells are both morphologically and biochemically differentiated.

## Results and discussion

### Optimisation of matrices for differentiation of SH-SY5Y cells

**Figure 1:**
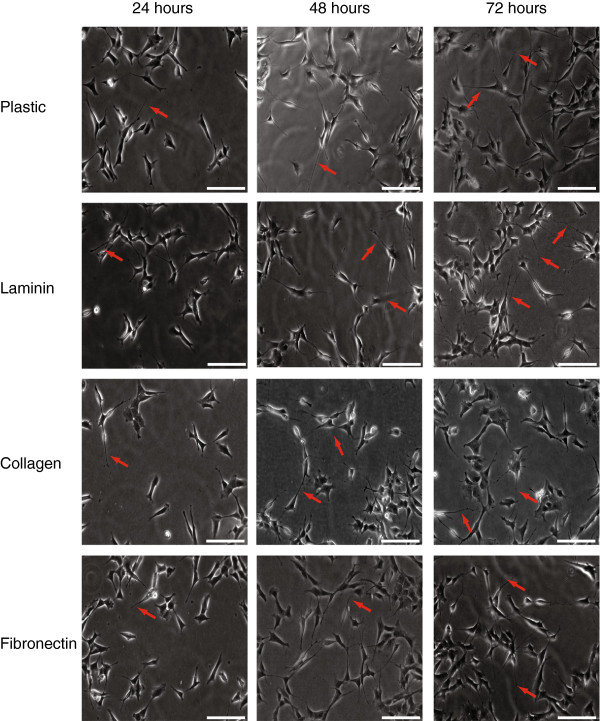
**Optimisation of matrices for differentiation of SH-SY5Y cells.** SH-SY5Y cells were plated on 6 well plates uncoated (plastic) or coated with 10 μg/ml of laminin, collagen or fibronectin. Cells were incubated in regular DMEM media containing 10% FBS for 24, 48 or 72 hours. Pictures were taken at 20× using Metamorph software. Scale bar = 20 μm.

**Figure 2:**
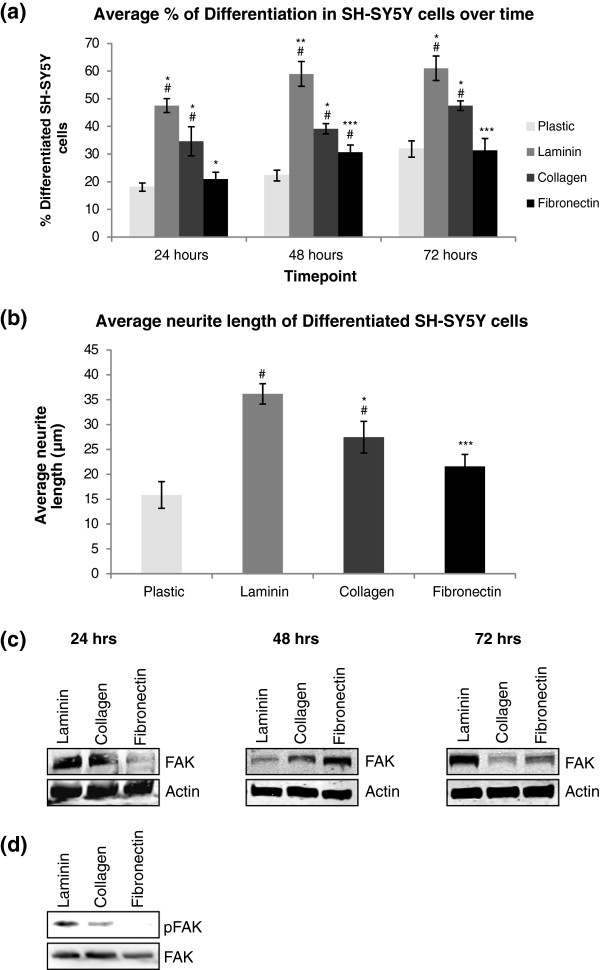
**Quantitative analysis of neurite outgrowth behaviour on different substrates. (a)** SH-SY5Y cells were plated on uncoated (plastic) or surfaces coated with 10 μg/ml of different ECM proteins: laminin, collagen or fibronectin. Cells were incubated in regular DMEM media containing 10% FBS for 24, 48 or 72 hours. Cells were counted from each condition at each timepoint and the number of differentiated cells was expressed as a percentage of the total cells counted ± SEM, n = 3. **(b)** The length of the neurites extending from the SH-SY5Y cells after 72 hours differentiation were measured and the average length for each matrix was expressed in a graph ± SEM, n = 3. Significant differences were measured by ANOVA (^#^P < 0.05 for comparisons between ECM protein-coated substrates and plastic; *P < 0.05 for comparisons between the coated substrates, Collagen vs. Laminin, Laminin vs Fibronectin or Collagen vs. Fibronectin). **(c)** Cells were lysed at each timepoint and run on 12% SDS-PAGE gels and probed for focal adhesion kinase (FAK), followed by detection with LI-COR Odyssey™, to monitor effect of matrices and time on protein levels. **(d)** To monitor the effect of the matrices on total FAK phosphorylation, cells were grown on each of the matrices for 24 hours before lysing. FAK was immunoprecipitated and the immunoprecipitate was run on 12% SDS-PAGE gel and probed for phospho-tyrosine and FAK followed by detection using the LI-COR Odyssey™ infrared image scanner.

Our first objective was to optimise a method to differentiate SH-SY5Y cells into a neuronal model cell line. Differentiation of cell lines into neuronal-like cell lines is required to mimic the intracellular environment of a neuronal cell. During differentiation, SH-SY5Y cells stop proliferating, extend long neurites and express neuron-specific markers, e.g. GAP43 and β3-tubulin. After differentiation, SH-SY5Y cells resemble dopaminergic neurons and can be used as a cell model for neurite outgrowth. Ivankovic-Dikic *et al.*[22] reported that both the adhesion and growth factor receptors need to be stimulated simultaneously in order for SH-SY5Y cells to undergo differentiation. In order to determine which component of the ECM would contribute to enhanced neurite outgrowth and FAK expression, SH-SY5Y cells were plated on uncoated 6 well plates or plates coated with 10 μg/ml of the extracellular matrix proteins: fibronectin, collagen and laminin. Cells were grown in DMEM supplemented with 10% FBS on these matrices for 72 hours, with media changed after 48 hours. Pictures were taken at 24, 48 and 72 hours to monitor neurite outgrowth (Figure 1). As differentiation is a time dependent and progressive event, our measurement of differentiation involved monitoring the cells over a 72 hour period to detect cessation of proliferation and extension of long neurites. The length of neurites were measured using Metamorph software and a differentiated cell was defined as a cell with a neurite length greater than the length of the cell body (on average greater than 10 μm in length). SH-SY5Y cells extended neurites on all three matrices with the longest and highest number of neurites found on cells plated on laminin for 72 hours (Figure 2 (a), (b)). Cells were lysed at the same interval times and run on an SDS-PAGE gel to monitor protein expression on the different matrices (Figure 2 (c)). SH-SY5Y cells also expressed high levels of FAK after 72 hours on laminin. The highest levels of phosphorylated FAK were detected after plating cells on laminin (Figure 2 (d)). For this reason, growth of SH-SY5Y cells on laminin for 72 hours was chosen for further study. During the course of this study, another group published the effects of different matrices on neuronal differentiation of the PC12 cell line [27]. This group also concluded that laminin induces higher levels of FAK expression and longer neurites. It is not surprising that laminin was found to induce the highest level of neurite outgrowth. Laminins are a major type of glycoprotein present in the ECM in the developing brain and stimulate neurite outgrowth in many neuronal cells in vitro [28,29].

### Optimisation of growth factor media for differentiation of SH-SY5Y cells

**Figure 3:**
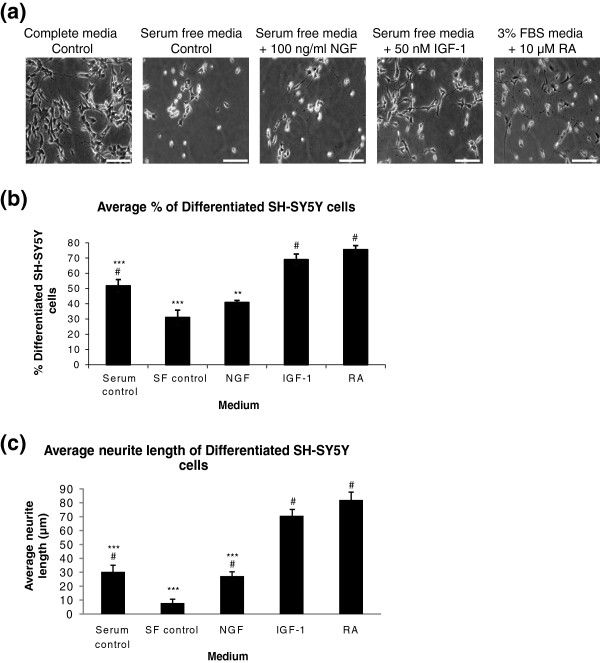
**Optimisation of growth factor media for differentiation of SH-SY5Y cells. (a)** SH-SY5Y cells were plated on 6 well plates coated with laminin and incubated in regular DMEM media containing 10% FBS (Complete media Control), serum free DMEM (Serum free media Control), serum free media containing 100 nM NGF, serum free media containing 50 nM IGF-1 or DMEM containing 3% FBS and 10 μM RA for 72 hours. Pictures were taken using Metamorph software. Scale bar = 50 μm **(b)** Cells were counted from each condition and the number of differentiated cells was expressed as a percentage of the total cells counted ± SEM, n = 3. **(c)** The length of the neurites extending from the SH-SY5Y cells after 72 hours differentiation were measured and the average length for each media was expressed in a graph ± SEM, n = 3. Significant differences were measured by ANOVA (^#^P < 0.05 for comparisons between serum free media and all other treatments; *P < 0.05 for comparisons between RA media and all other treatments.

Next, we needed to optimise the growth factor required for differentiation of SH-SY5Y. Having determined that growth of SH-SY5Y cells on laminin for 72 hours induces highest levels of FAK expression and neurite outgrowth, we were interested in determining which growth factor/chemical stimulates the highest level of neurite outgrowth. NGF is commonly used to differentiate PC12 cells and has been the optimal choice for many years [30]. However, the choice of medium required for differentiation of SH-SY5Y cells has not been defined. Previous studies have shown that SH-SY5Y cells can be differentiated using retinoic acid [31,32], phorbol esters such as 12-O-tetradecanoyl-phorbol-13-acetate (TPA) [33,34] and growth factors including IGF-1 and BDNF [22,35,36]. In order to stimulate cell receptors, SH-SY5Y cells were plated on laminin coated 6 well plates and stimulated with a range of growth factor containing media: complete DMEM media containing 10% FBS, serum free DMEM, serum free DMEM containing 100 ng/ml NGF (to stimulate TrkA receptor), serum free DMEM containing 50 nM IGF-1 (to stimulate IGF-1R) and DMEM containing 3% FBS and 10 μM RA (to stimulate retinoid acid nuclear receptors). Complete media was used as a control for stimulation of multiple growth factor receptors as complete media contains growth factors such as EGF [37,38], PDGF [39,40], FGF [41], NGF [42], IGF-1 and IGF-2 [43]. Serum free media was used as a control for lack of any growth factor stimulation. Cells were grown in these conditions for 72 hours, with media changes after 48 hours. Neurite outgrowth was monitored over time and pictures were taken at 72 hours (Figure 3 (a)). Cells were counted from each condition and the number of differentiated cells was expressed as a percentage of the total cells counted (Figure 3 (b)). The morphological differentiation of neuronal cells involves arrest of proliferation and extension of long neurites. Neurite outgrowth (differentiation) was defined as cells with neurites longer than one cell body length. As PC12 cells require NGF for differentiation, it was surprising to find that NGF induced only low levels of neurite outgrowth in SH-SY5Y cells. However, our observation is supported by previous data. Recio-Pinto *et al.* (1984) reported that NGF (via stimulation of the TrkA receptor) does not enhance neurite outgrowth in SH-SY5Y cells cultured under serum free conditions [44,45]. SH-SY5Y cells had the highest levels of neurite outgrowth and longest neurites after stimulation with 10 μM RA for 72 hours (Figure 3 (c)). However, there was no significant difference between stimulation with RA and stimulation with 50 nM IGF-1 for 72 hours (Figure 3 (b),(c)). For this reason, both treatments were evaluated further in this study to ensure the cells were biochemically differentiated to mimic the intracellular environment of a neuronal cell.

### Confirmation of biochemical differentiation of SH-SY5Y cells

**Figure 4:**
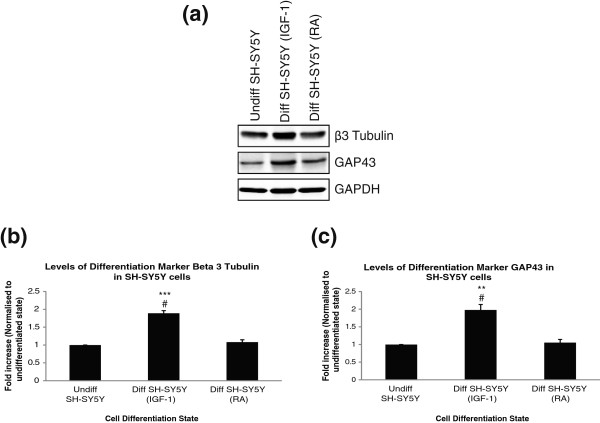
**Confirmation of biochemical differentiation of SH-SY5Y. (a)** Lysates of SH-SY5Y cells, undifferentiated or differentiated with IGF-1 or RA were run on 12% SDS-PAGE gels and probed for differentiation marker proteins, β3 tubulin and GAP43, followed by detection with LI-COR Odyssey™. Densitometry of protein bands was measured using LI-COR Odyssey™ software and normalised to cellular GAPDH levels. **(b)** The fold increase in β3 tubulin signal compared to undifferentiated protein level are plotted on a bar chart ± SEM, n = 3. **(c)** The fold increase in GAP43 signal compared to undifferentiated protein level are plotted on a bar chart ± SEM, n = 3. Significant differences were measured by ANOVA (^#^P < 0.05 for comparisons between undifferentiated cells and both differentiation conditions; *P < 0.05 for comparisons between RA differentiated cells and IGF-1 differentiated cells.

Having determined that the SH-SY5Y cell line was morphologically differentiated with treatment with either RA or IGF-1, it was next important to confirm that the cell lines were also biochemically differentiated. Differentiated neuronal cells express higher levels of neuronal specific markers, β3 tubulin and GAP43 [46-50]. SH-SY5Y cells were plated on laminin in either complete DMEM containing 10% FBS (undifferentiated), serum free DMEM containing 50 nM IGF-1 for 72 hours (differentiated IGF-1) or DMEM containing 3% FBS and 10 μM RA (differentiated RA). Cells were lysed and run on an SDS-PAGE gel to monitor protein expression of neuronal markers before and after differentiation. Densitometry of protein bands was measured using LI-COR Odyssey™ software and the fold increase in signal compared to undifferentiated protein level plotted on a bar chart. As shown in Figure 4, while the undifferentiated SH-SY5Y cells did express both β3 tubulin and GAP43, the level of both proteins was higher after differentiation with IGF-1 but not RA. This confirms the SH-SY5Y cells are biochemically differentiated only when treated with IGF-1. Many studies have reported the use of retinoic acid to differentiate SH-SY5Y [31,36,51,52]. Retinoic acid is a cheaper option for differentiation compared to use of growth factors. However, although the cells were morphologically differentiated, we found that they were not biochemically differentiated and were therefore unsuitable for our study. We determined that the optimal conditions to differentiate SH-SY5Y cells into a neuronal model cell line that is morphologically and biochemically different than undifferentiated cells are incubation for 72 hours on laminin in serum free DMEM with 50 nM IGF-1.

### Confirmation of differentiation parameters for SH-SY5Y cells

**Figure 5:**
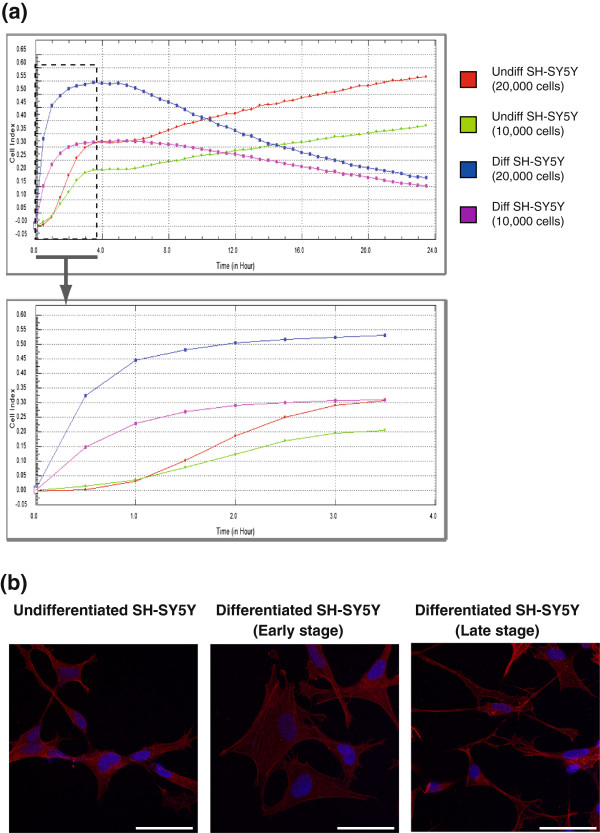
**Confirmation of differentiation parameters for SH-SY5Y cells. (a)** SH-SY5Y cells were plated at concentrations of 10,000 cells or 20,000 cells per well in an xCELLigence E-plate (ROCHE) in regular DMEM media containing 10% FBS (undifferentiated cells) or in serum free DMEM containing 50 nM IGF-1 (differentiated cells). The xCELLigence system measures changes in impedance as cells attach with a readout given as cell index (CI) value. The baseline impedance is recorded using control wells containing DMEM only with no cells. Graph representative of duplicate wells and shows adhesion and proliferation over 24 hours, n = 2. A zoomed area of the first 3.5 hours shows differentiated cells attach earlier and are larger but do not proliferate. **(b)** SH-SY5Y cells were plated on laminin-coated glass coverslips and incubated in regular DMEM media containing 10% FBS (undifferentiated cells) or in serum free DMEM containing 50 nM IGF-1 (differentiated cells). Cells were fixed with 4% PFA, permeabilised with PHEM/0.1% Triton X, blocked with PHEM/5% goat serum. The actin cytoskeleton was stained with TRITC-phalloidin (red) and the nuclei were stained with Hoechst 33242 (blue). All images were acquired sequentially at 63× and images merged using ImageJ. Scale bar = 20 μm. Early stage differentiated cells are larger than undifferentiated cells but cell body size decreases as neurites extend in late stage differentiation. This supports the pattern seen in the xCELLigence graph.

Having determined that growth of SH-SY5Y cells on laminin for 72 hours in serum free DMEM containing 50 nM IGF-1 induces highest levels of FAK expression and highest level of neurite outgrowth, we wanted to confirm that this cell model could be used to study changes in cell adhesion and migration. In order to test this, SH-SY5Y cells were incubated in either complete DMEM media with 10% FBS (undifferentiated population) or serum free DMEM with 50 nM IGF-1 (differentiated population) at a concentration of either 1.0 or 2.0 × 10^4^ cells on laminin coated E-plate wells and placed in the xCELLigence system (Roche). The xCELLigence system measures changes in impedence as cells attach with a readout given as cell index (CI) value and monitors cell behaviour in real time. An increase in the number of cells attaching, an increase in cell size or an increase in the strength of adhesion results in an increased CI value. The baseline impedence is recorded using control wells containing DMEM only with no cells. The impedence of the cells in culture for each condition was measured every 30 minutes for 24 hours and expressed as a CI value. As shown in Figure 5 (a) and as expected, the higher number of cells plated (20,000) gave a higher CI value than the lower number (10,000) as more cells were attaching to the gold plated surface and affecting the impedence. Unexpectedly, the differentiated cells appear to adhere quicker to the surface than undifferentiated cells; however, the impedence falls off rapidly as they are not proliferating. This is explained by observations we have made about the early processes of differentiation. SH-SY5Y cells were plated on laminin coated glass coverslips and incubated in complete DMEM (undifferentiated) or serum free DMEM containing IGF-1 (differentiated). Cells were fixed at varying points throughout the differen-tiation process and stained with TRITC-phalloidin to visualise the actin cytoskeleton and Hoechst 33342 to visualise the nuclei (Figure 5 (b)). During the initial stages of SH-SY5Y differentiation, the cell body becomes larger and later, the cells begin to extend neurites and the cell body shrinks in size. This could explain the graph from the xCELLigence as the cells may adhere quicker, the cell body may extend in size (causing as increase in impedence and consequently, an increase in cell index) but after the extension of neurites, the cell body shrinks and the impedence (and cell index) drops. Using this live cell monitoring system, we clearly separated and defined the two unique populations of SH-SY5Y cells (undifferentiated and differentiated). The optimal conditions described produce cell populations that would be useful for monitoring cell adhesion and migration.

### Immunofluorescent staining of activated FAK in SH-SY5Y cells

**Figure 6:**
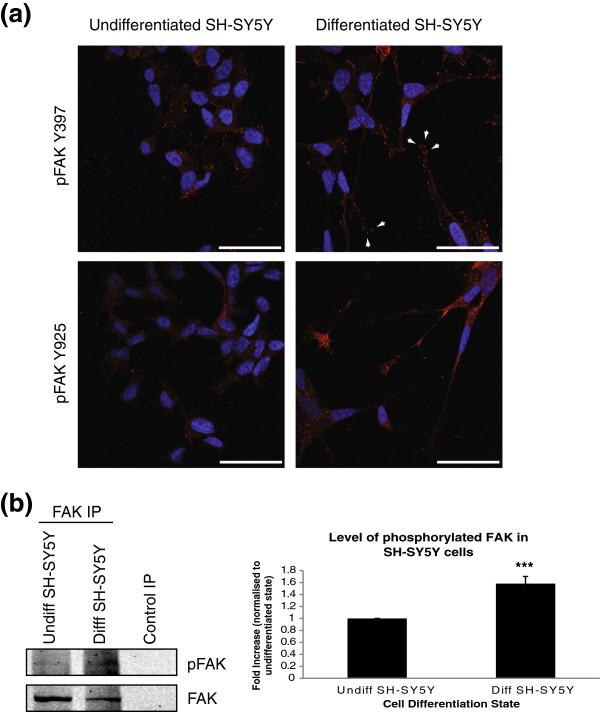
**(a) Immunofluorescent staining of activated FAK in SH-SY5Y cells.** SH-SY5Y cells were plated on laminin coated coverslips in complete DMEM (undifferentiated) or differentiated in serum free DMEM with IGF-1 for 72 hours. Cells were fixed in 4% paraformaldehyde, permeabilised in PHEM/0.1% Triton X and blocked in PHEM/5% goat serum. Cells were stained with either pFAK Y^397^ or pFAK Y^925^ (red) and the nuclei were stained with Hoechst 33242 (blue). All images were acquired sequentially at 63× and images merged using ImageJ. Scale bar = 20 μm. **(b)** FAK was immunoprecipitated from lysates of SH-SY5Y cells, undifferentiated or differentiated with IGF-1 and run on 12% SDS-PAGE gels and probed for phospho-tyrosine and FAK, followed by detection with LI-COR Odyssey™. Densitometry of protein bands was measured using LI-COR Odyssey™ software and normalised to undifferentiated levels. The fold increase in pFAK signal compared to undifferentiated protein level are plotted on a bar chart ± SEM, n = 3.

Having determined the optimal conditions for differentiation of SH-SY5Y cells, we wanted to ensure we could monitor changes in cell morphology and protein localisation in these cells. SH-SY5Y cells were plated on laminin-coated coverslips in serum free DMEM containing 50 nM IGF-1 for 72 hours. Cells were fixed with 4% paraformaldehyde, permeabilised in PHEM/0.1% Triton X and blocked in PHEM/5% goat serum. Cells were incubated with primary antibody against activated FAK, either pFAK Y^397^ or pFAK Y^925^ and a Cy3 secondary antibody (red) was used. The nuclei were stained with Hoechst 33242 (blue). The tyrosine residue at position 397 becomes phosphorylated during focal adhesion formation, while phosphorylation of tyrosine residue 925 of FAK is required during focal adhesion turnover. An increase in both adhesion formation and turnover are required for effective neurite outgrowth. As shown in Figure 6 (a), the end of the neurites from differentiated SH-SY5Y cells contain growth cones containing high levels of phosphorylated (active) FAK and high numbers of focal adhesions (arrowheads) confirming previous reports that FAK is active in growth cones of neurons and is required for neurite outgrowth [11,53]. As shown in Figure 6 (b), stimulation of SH-SY5Y cells with IGF-1 to induce differentiation leads to a significant increase in the overall levels of phosphorylated FAK. This confirms that the SH-SY5Y model described here is a good model to study the molecular events underlying cell adhesion and cell migration and has potential for studying changes in protein localisation or protein-protein interactions after differentiation.

## Conclusions

In this study, we demonstrate an optimised protocol for obtaining a morphologically and biochemically distinct population of a neuronal model cell line, SH-SY5Y. These cells have a distinct adhesion and spreading pattern when compared to undifferentiated SH-SY5Y cells. The protocol has been optimised for FAK protein expression, a protein that plays an integral part in orchestrating focal adhesion assembly downstream of growth factor and adhesion receptor signalling. This protocol will be useful for researchers that are studying neuronal protein-protein interactions but are unable to use or manipulate primary hippocampal neurons.

## Methods

### Reagents and antibodies

Recombinant NGF was purchased from R&D Systems Europe Ltd. (Abington, UK). Recombinant IGF-1 was purchased from Peprotech Inc. (Rocky Hill, NJ). All trans retinoic acid was purchased from Sigma-Aldrich (Wicklow, IE). Laminin was purchased from Miltenyi Biotech (Surrey, UK). Collagen and fibronectin were purchased from Sigma-Aldrich (Wicklow, IE). The rabbit anti-FAK antibody and anti-phospho FAK Y397 were from Santa Cruz Biotechnology (Santa Cruz, CA). The rabbit anti-GAP43 antibody was purchased from Millipore (Cork, IE). GAPDH was from Advanced Immunochemical Inc. (Long Beach, CA). Mouse anti-β3 tubulin antibody was purchased from Promega (Southampton, UK). Anti-phospho FAK (Tyr-925) polyclonal antibody was from Cell Signalling Technology (Beverly, MA).

Secondary antibodies were purchased from LI-COR (Cambridge, UK) (western blotting) and Jackson Immunoresearch (Suffolk, UK) (for immunofluorescence).

### Cell culture and differentiation of SH-SY5Y cells

The human neuroblastoma cell line, SH-SY5Y was purchased from ATCC and maintained in Dulbecco’s modied Eagle’s medium (DMEM) (Sigma-Aldrich Ltd.) supplemented with 10% (v/v) foetal bovine serum (FBS), 10 mM L-Glutamine, and 5 mg/ml penicillin/streptomycin. To optimise matrices for differentiation, SH-SY5Y cells were plated on 10 μg/ml laminin, collagen or fibronectin and incubated in DMEM for 24, 48 and 72 hours to induce differentiation. Medium was replenished after 48 hours. To optimise growth factor for differentiation, SH-SY5Y cells were plated on 10 μg/ml laminin incubated in complete DMEM, serum-free DMEM, serum-free medium containing NGF, serum-free medium containing IGF-1 or DMEM containing 3% FBS and 10 μM all-trans retinoic acid (RA) for 72 hours to induce differentiation. Differentiation medium was replenished after 48 hours. The morphological assessment of differentiation involved monitoring the cells over a 72 hour period to detect the extension of long neurites from the cell body. Metamorph™ software was used to measure the length of individual neurites on captured images. A differentiated cell was defined as a cell with a neurite length greater than the cell body of the individual cell (on average greater than 10 μm in length).

### Preparation of cellular protein extracts

Cellular protein extracts were prepared by washing cells in ice cold PBS and scraping into ice cold lysis buffer (10 mM Tris HCl pH 7.4, 150 mM NaCl, NaF, 1% NP40 plus the tyrosine phosphatase inhibitor Na3VO4 (1 mM), protease inhibitors PMSF (1 mM), pepstatin (1 μM) and aprotinin (1.5 μg/ml)). Lysates were incubated on ice for 20 minutes before centrifugation at 14,000 rpm for 15 minutes at 4°C to remove nuclei and cellular debris. Lysates were analysed for protein concentration using the Bradford assay and boiled in sample buffer for SDS-PAGE.

### Immunofluorescence to detect protein localisation in cells

SH-SY5Y cells were seeded at 5×10^4^ and differentiated on laminin coated 10 mm glass coverslips. The cells were fixed with 4% paraformaldehyde for 1 hour and permeabilized by incubation in PHEM/ 0.1% Triton X-100 for 15 min. The cells were rinsed three times in PHEM and blocked with 5% of normal goat serum (NGS) for 30 min and finally incubated for 2 hours at room temperature with primary antibodies (pFAK Y^397^ or Y^925^). The cells were then incubated for 1 hour at room temperature with the dye-labeled secondary antibody Cy3 (red) and Hoechst 33342 (stains nuclei). For studies on actin structure of cells, TRITC phalloidin was added to cells along with Hoechst 33342 after blocking. Coverslips were mounted using Vinol (Sigma) and cells were examined with a Zeiss LSM 710 META confocal laser scanning microscope equipped with an argon/krypton laser. All experiments were analyzed in sequential scanning mode. Images (1024 × 1024 pixels) were obtained with a × 63 magnification oil-immersion objective.

### Cell adhesion and cell spreading assay

E-plates (16 wells) were coated with laminin (10 μg/ml) in serum-free DMEM for 30 mins at 37°C and washed twice with PBS. SH-SY5Y cells were harvested with trypsin/EDTA, washed with DMEM, and resuspended in either complete DMEM media with 10% FBS or differentiation media (serum free DMEM with 50 nM IGF-1) to give a final density of 1.5× 10^5^ cells/ml. This cell suspension (100 μl/1.5 × 10^4^ cells) was plated onto laminin-coated E-plates and placed in the xCELLigence system (Roche). The xCELLigence system measures changes in impedence as cells attach with a readout given as CI value. The baseline impedence is recorded using control wells containing DMEM only with no cells. Scans were run for 24 hours with sweeps every 30 minutes to detect early stages of cell attachment.

## Abbreviations

IGF-1: Insulin-like growth factor-1; NGF: Nerve growth factor; DMEM: Dulbecco’s modied Eagle’s medium; RA: Retinoic acid.

## Competing interests

The authors declare that they have no competing interests.

## Authors’ contributions

SD designed and performed experiments and drafted the manuscript. ED performed experiments. PAK supervised the study, conceived the study design, designed the manuscript, performed manuscript critical review and revised the draft manuscript. All authors read and approved the final manuscript.
